# Physical Therapy Approaches for Concomitant Anterior Cruciate Ligament and Posterior Cruciate Ligament Avulsion Fractures: A Case Report

**DOI:** 10.7759/cureus.69959

**Published:** 2024-09-22

**Authors:** Maitri V Thamke, Swapnil U Ramteke, Ashish Keoliya

**Affiliations:** 1 Physiotherapy, Ravi Nair Physiotherapy College, Datta Meghe Institute of Higher Education & Research, Wardha, IND; 2 Sports Physiotherapy, Ravi Nair Physiotherapy College, Datta Meghe Institute of Higher Education & Research, Wardha, IND

**Keywords:** acl, avulsion fracture, faradism under pressure, mcl, pcl, physical therapy

## Abstract

Multi-ligament knee injury, involving complex destruction of structures like the anterior cruciate ligament (ACL) and posterior cruciate ligament (PCL), presents significant challenges in treatment as well as diagnosis. This case study focuses on a 55-year-old woman who was the victim of a severe traffic accident and had an ACL and PCL avulsion fracture, a medial condyle fracture, and a medial collateral ligament rupture. Afterward, open reduction and internal fixation of the right tibial plateau surgery was performed after a delay in order to manage the pain and swelling. Her rehabilitation was greatly helped by postoperative physical therapy, which focused on pain control, early mobilization, and gradual strength development. A well-structured rehabilitation plan was found to be beneficial, as indicated by the remarkable improvements in pain reduction and functional mobility. This case highlights the value of effective physical therapy in helping patients through the difficult recovery process following multiple ligament injuries with the goal of restoring function and improving their quality of life.

## Introduction

Multi-ligament knee injury (MLKI) involves multiple major knee ligaments, including the anterior cruciate ligament (ACL), posterior cruciate ligament (PCL), medial collateral ligament (MCL), and lateral collateral ligament (LCL), causing significant pain and swelling [1]. The proximal tibia is the point of origin of the ACL, which is responsible for sagittal stability and for preventing the posterior translation of the femur on the tibia during weight-shifting activities like stair-climbing [2]. The ACL and the PCL cross at the lateral aspect of the medial femoral condyle. Although it can strain in specific positions, such as climbing stairs, its primary function is to prevent anterior shifting of the distal femur and posterior migration of the proximal tibia [3]. The MCL lies between the space of the distal femur and the proximal tibia [4]. Between the proximal tibia and distal femur, the LCL and IT band work together to prevent varus strain on the knee [5].

The knee joint involved in multiple ligament injuries and fractures are rare but severe complications that can occur due to sports involving physical contact or severe car crashes. Research has revealed a delayed detection of patients with ligamentous injuries who have femoral or tibial injuries on the same side [6]. MRI is the best modality in diagnosing MLKIs, mainly because it shows the different ligaments that are injured [7]. Patients with MLKIs may complain of excessive pain and numbness in the leg or foot, which may indicate neurovascular involvement [8]. The multi-ligament injured knees are a challenging problem in orthopedic surgery, as they involve tears of the ACL and PCL with one of the collateral ligaments [9]. Literature reviews show that ligamentous injuries are diagnosed after a considerable time when individuals arrive with ipsilateral tibial or femur fractures [10].

It is essential to fix the condyle fracture and address the ligamentous instability as they contribute to the onset of osteoarthritis following traumatic injury. This can compromise the overall functional outcome due to pain and stiffness because they involve the articular surface [11]. Knee injuries can lead to significant morbidity, severe discomfort, and instability continuing for years after the original damage [12]. Knee dislocation, involving multiple ligament disruptions and neurovascular damage, is associated with significant morbidity. The treatment of MLKI is complex, leading to high complication rates [11]. Physiotherapy is crucial for orthopedic surgery intervention, focusing on early mobilization and rehabilitation. It is advised before surgery to maximize knee functions and after surgery to reduce recovery time [13]. Presurgical exercise programs can significantly improve patient outcomes for knee surgery. By enhancing knee range of motion (ROM), strengthening key muscles, and identifying potential candidates for noninvasive treatments, these programs offer a more personalized and effective approach to recovery. This, in turn, has enabled healthcare providers to make more informed decisions about rehabilitation timelines, ultimately leading to better patient experiences [14]. The goal of physiotherapy is to gradually reduce pain, increase the ROM, and strengthen joint muscles using electrical therapy, hydrotherapy, physical massage, and active exercise [15].

## Case presentation

A 55-year-old woman arrived at the emergency department following a road traffic accident (RTA) on July 4th, in which a four-wheeler crashed into a tree. She had been sitting in the passenger seat and was struck on the right lower limb by the dashboard. The patient reported acute pain and swelling in her right knee, which progressively worsened. She was unable to move her limb. There was no history of head injury, loss of consciousness, seizures, or ENT bleeding.

Initially, the patient was taken to a private hospital, where two stab wounds were cleaned and sutured. As part of primary management, an above-knee slab was applied, and MRI and X-rays were performed. She had no history of associated conditions like tuberculosis, hypertension, or diabetes mellitus.

Due to the nature of the RTA, the patient was referred to Acharya Vinoba Bhave Rural Hospital, Sawangi, in the first week of July. Radiological investigations revealed an ACL and PCL avulsion fracture with an MCL tear on the right side. The operation was delayed because of significant swelling. Subsequently, an open reduction and internal fixation (ORIF) with a 28-mm Herbert cannulated cancellous screw in an anteroposterior fashion was performed in the fourth week of July. The patient was referred for physiotherapy pre-rehabilitation for three weeks. Rehabilitation, both before and after surgery, was critical for recovery. She began active movements of the operated knee on day one post-ORIF to prevent arthrofibrosis and stiffness.

Clinical findings

The patient was evaluated after obtaining written consent. She was conscious, cooperative, and oriented to person, time, and place. The assessment was conducted while she lay supine, with her right leg elevated on pillows to reduce swelling. The patient reported dull, aching pain in her right knee, which suddenly intensified with movement. Upon examination, her Visual Analogue Scale scores indicated 9.1/10 during activity and 4.3/10 at rest. Local examination revealed grade II tenderness (the patient complained of pain and winced) in the right knee.

Due to pain and swelling, the pre-treatment range of motion (ROM) and manual muscle testing (MMT) were not in full range, as indicated by the negative findings in MMT. The physical examination included MMT and ROM assessments (Table 1, Table 2).

**Table 1:** MMT results pre- and post-intervention MMT, manual muscle testing

Movement	Pre-treatment: right knee (affected)	Post-treatment: right knee (affected)	Pre-treatment: left knee (non-affected)	Post-treatment: left knee (non-affected)
Hip flexors	-3/5	4/5	5/5	5/5
Hip extensors	-3/5	4/5	5/5	5/5
Hip adductors	-3/5	4/5	5/5	5/5
Hip abductors	-3/5	4/5	5/5	5/5
Knee flexors	1/5	2/5	5/5	5/5
Knee extensors	1/5	2/5	5/5	5/5
Ankle plantar flexors	-3/5	3/5	5/5	5/5
Ankle dorsiflexors	-3/5	3/5	5/5	5/5

**Table 2:** ROM ROM, range of motion

Action	Pre-treatment: right knee (affected)	Post-treatment: right knee (affected)	Pre-treatment: left knee (non-affected)	Post-treatment: left knee (non-affected)
Hip flexion	0-30°	0-50°	0-110°	0-110°
Hip extension	0-20°	0-25°	0-25°	0-30°
Hip adduction	0-10°	0-25°	0-25°	0-30°
Hip abduction	0-20°	0-40°	0-40°	0-45°
Knee flexion	0	0-40°	0-125°	0-130°
Ankle plantarflexion	0-10°	0-20°	0-40°	0-45°
Ankle dorsiflexion	0-10°	0-15°	0-15°	0-20°

Investigation

**Figure 1:**
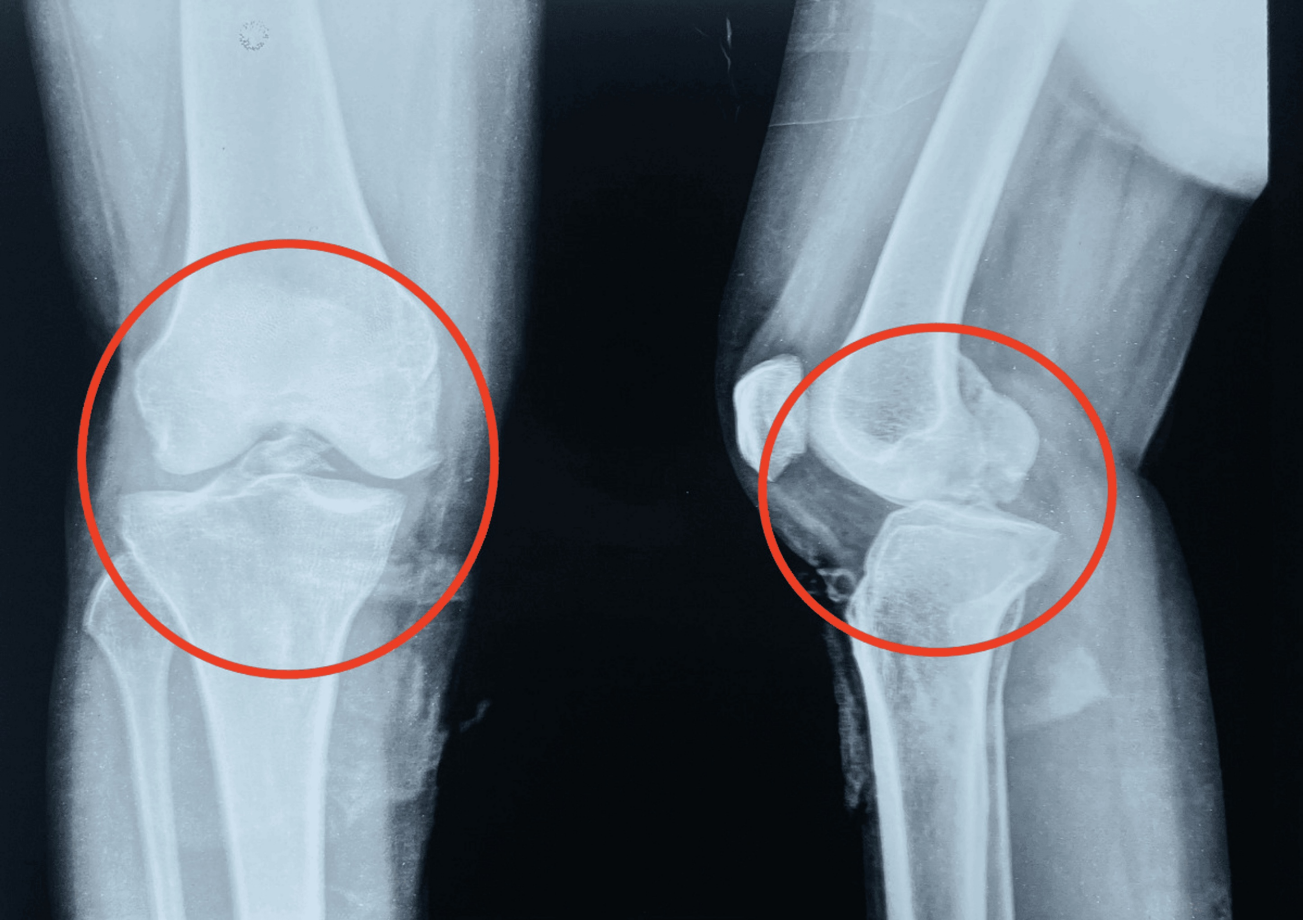
X-ray of the right knee in AP and lateral view showing a tibial condyle avulsion fracture

An X-ray of the right knee was performed, revealing an avulsion fracture of the tibial condyle (Figure 1). An MRI of the right knee joint was also conducted. The MRI findings indicated a fracture in the proximal part of the tibia, involving the tibial plateaus, predominantly the medial tibial plateau. An avulsion fracture of the ACL and PCL was observed at their tibial attachments, along with a high-grade tear of both ligaments near their tibial attachments. There was discontinuity of the MCL near its femoral attachment, suggestive of a grade 3 tear. Multiple osseous and ligamentous injuries were noted in the conclusion (Figure 1).

**Figure 2:**
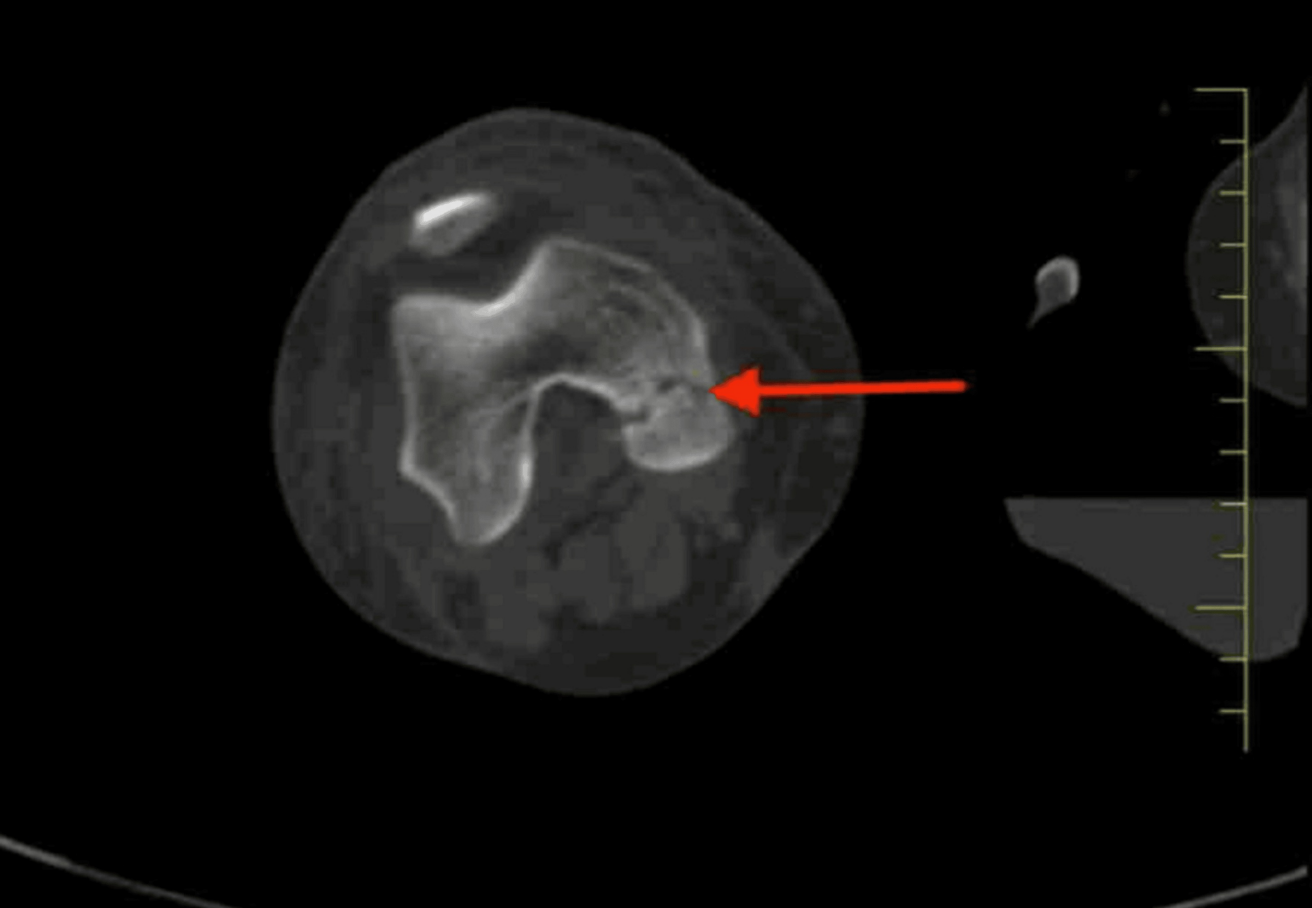
MRI scan showing avulsion fractures of the ACL and PCL at their tibial attachments ACL, anterior cruciate ligament; PCL, posterior cruciate ligament

Figure 2 demonstrates avulsion fractures of the ACL and PCL at the tibial site, as identified on an MRI scan.

**Figure 3:**
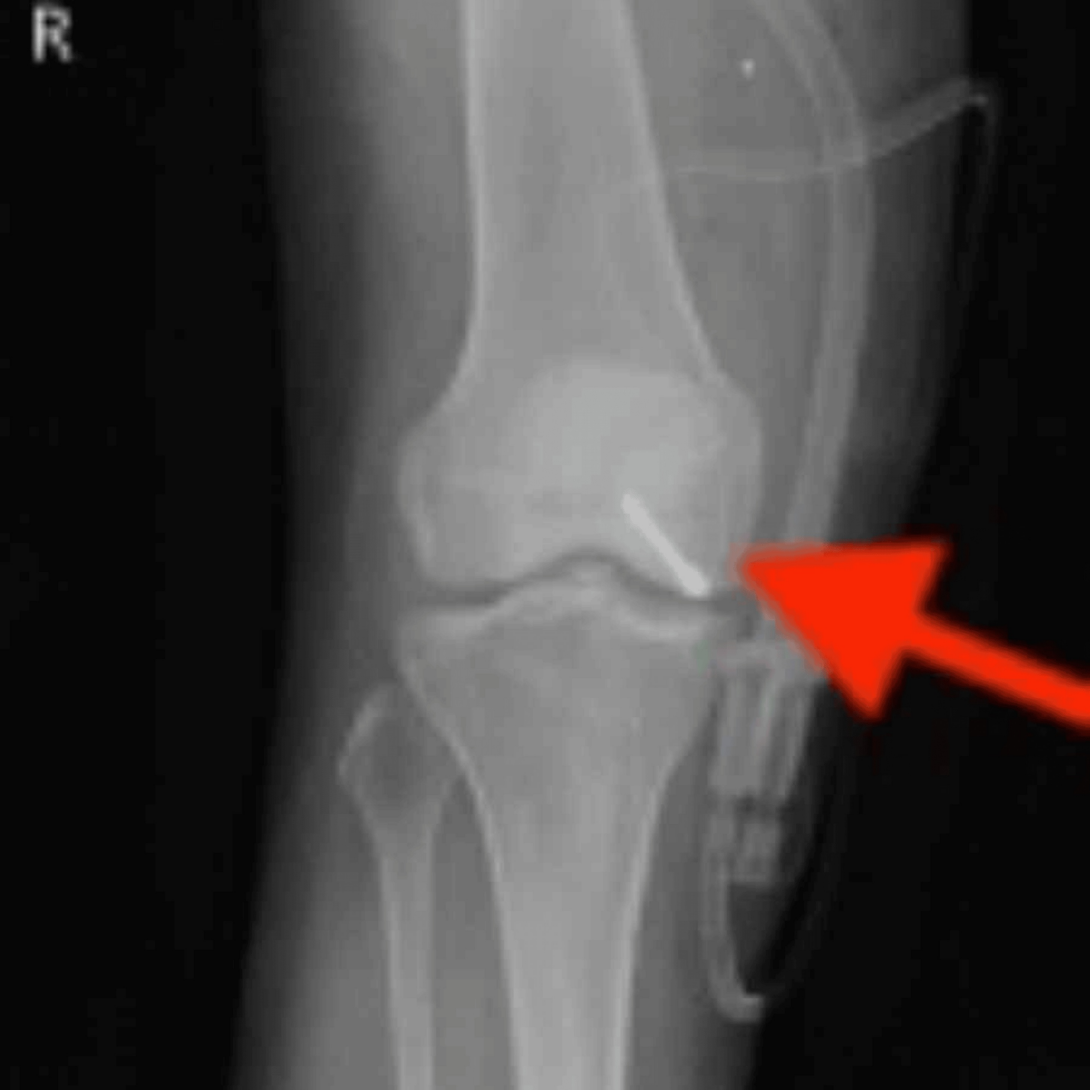
ORIF with CC screw (Herbert screw 28 mm XL) CC, cannulated cancellous; ORIF, open reduction and internal fixation

The postoperative X-ray demonstrates ORIF using Herbert screws (28 mm XL) oriented in an anteroposterior direction, as illustrated in Figure 3.

Physiotherapy intervention

The physiotherapy intervention was formulated based on the patient’s structural and functional impairments, as shown in Table 3, Table 4, Table 5, Table 6, and Table 7. It was formed according to the International Classification of Functioning, Disability and Health format.

**Table 3:** Structural impairment ACL, anterior cruciate ligament; PCL, posterior cruciate ligament

Structural impairment	Source of information	Clinical reason
ACL and PCL tear (right side)	MRI	Trauma
Medial tibial plateau fracture	X-ray	Trauma
Swelling around the knee joint	On observation	Because of fracture
Tenderness around the knee	On palpation	Because of inflammation

**Table 4:** Functional impairment MMT, manual muscle testing; ROM, range of motion

Functional impairment	Source of information	Clinical reason
Reduced muscle power	MMT	Disuse atrophy
Reduced mobility of the right knee	ROM	Pain
Pain at the medial and lateral aspects of the knee	Pain assessment	Trauma

**Table 5:** Activity limitations ROM, range of motion

Activity limitations	Source of information	Clinical reason
Walking	Assessment	Pain and reduced ROM
Challenges in mobility	Assessment	Challenges in moving, like climbing stairs
Lifting and carrying objects	Assessment	Due to balance impairment
All the daily living activities	Assessment	Due to pain and swelling

**Table 6:** Participation restriction ROM, range of motion

Participation restriction	Clinical reason
Unable to attend work (go to the farm)	Due to pain, reducing ROM and swelling
Unable to move around in different locations	Post-surgery limitations
Unable to attend a family function	Due to pain and swelling

**Table 7:** Contextual factors

Contextual factors	Clinical reason
Environmental factors	Need of crutches
	Need physical therapy
	Need for home modifications
Personal factors	Need psychological support
	Need family support
	Need financial support

The pre-rehabilitation program was planned to reduce the patient’s swelling and pain and was administered over three weeks. Following the operation, physiotherapy rehabilitation was provided for an additional three weeks, focusing on muscle strength, balance, and mobility. After the patient was informed about her condition, all precautions were taken to avoid unnecessary stress on the healing structures. The physiotherapy interventions were tailored to the patient’s pain limits. The goal was to enhance strength, balance, and flexibility through functional activities by gradually increasing sets, repetitions, or reducing the duration of physiotherapy sessions. Table 8 presents a breakdown of the physiotherapy treatments administered during the first week after the operation.

**Table 8:** Day 1 to week 1 DVT, deep vein thrombosis; ROM, range of motion

Goals	Intervention	Rationale	Repetitions
To educate the patient about injury management and precautions	Inform the patient and her family of the injury’s severity and the estimated period of time needed for recovery. Teach the use of a walker for gait training. Explain the importance of physiotherapy in her present condition.	Education guarantees adherence to the rehabilitation program and gives the patient the confidence to participate actively in her own healing.	-
To use a knee brace for maximum protection	It is used to prevent knee stiffness and initiate healing.	Using a knee brace and walker to protect the knee joint reduces stress on the injured parts and promotes appropriate healing of the fracture.	Whenever movement is initiated
To reduce pain	Rest icing compression elevation	Used in acute stage	Apply the ice pack for 10-20 minutes, three or more times a day.
To reduce swelling	All-time limb elevation	Elevation facilitates the reduction of swelling	All day
To increase bed mobility	Bedside sitting	Increases mobility and improves functional independence	Twice a day
To increase circulation and prevent DVT	Bilateral ankle-toe movements	Ankle-toe movements increase blood circulation and prevent secondary complications like deep vein thrombosis.	10 reps × 1 set
To increase mobility	Active ROM exercises	Active ROM increases the mobility of the limbs	10 reps × 1 set
Muscle re-education	Faradism under pressure	In the early stages of non-weight bearing, it helps in maintaining muscle function and preventing muscle wasting.	10 minutes

Table 9 outlines the physiotherapy treatments administered during the second, third, and fourth weeks following the operation.

**Table 9:** Weeks 2-4 ROM, range of motion

Goals	Intervention	Rationale	Repetitions
To reduce pain and swelling	Continue the cryotherapy	Maintaining pain control is essential to the patient’s comfort and participation in the rehabilitation program.	Apply the ice pack for 10-20 minutes, three or more times a day.
To increase general strength	Single leg raise	Maintain stability	10 reps × 1 set
To restore movement and function	Passive ROMs	It prevents stiffness and maintains joint mobility	10 reps × 1 set
To prevent muscle atrophy and activate the muscle	Isometric exercises	These movements build and activate muscles without putting unwanted strain on the joint, preparing the body for strengthening exercises.	10 reps × 1 set
To initiate weight bearing	Weight bearing increased gradually as tolerated, progressing from a walker to a cane.	Progressive weight-bearing encourages continuous healing and response by managing stress on the healing bone and ligament.	Twice a day
To restore balance	Balance exercises on a flat surface	Regaining balance helps in restoring joint stability and reducing the risk of falling	Twice a day

**Figure 4:**
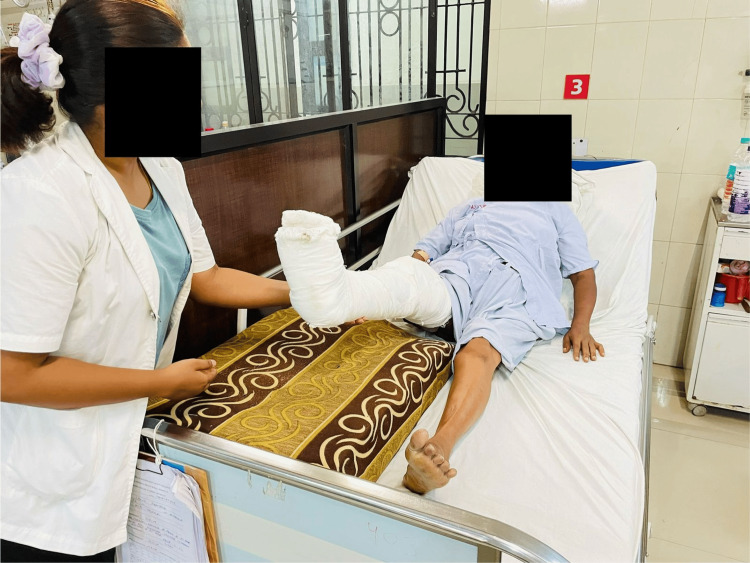
Single leg raise

Figure 4 illustrates an assisted single leg raise being performed.

**Figure 5:**
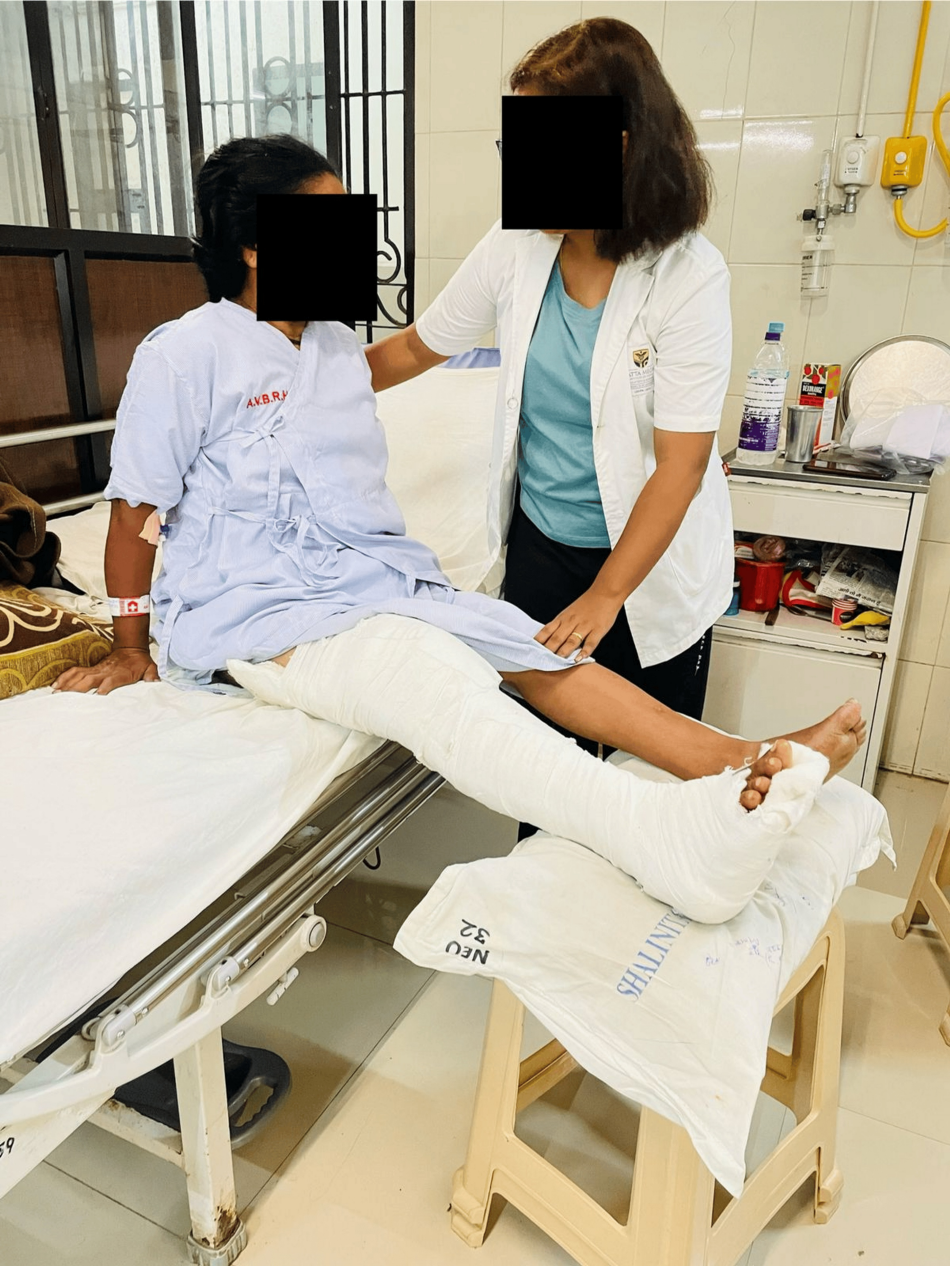
Bedside sitting

Figure 5 demonstrates assisted bedside sitting being performed.

**Figure 6:**
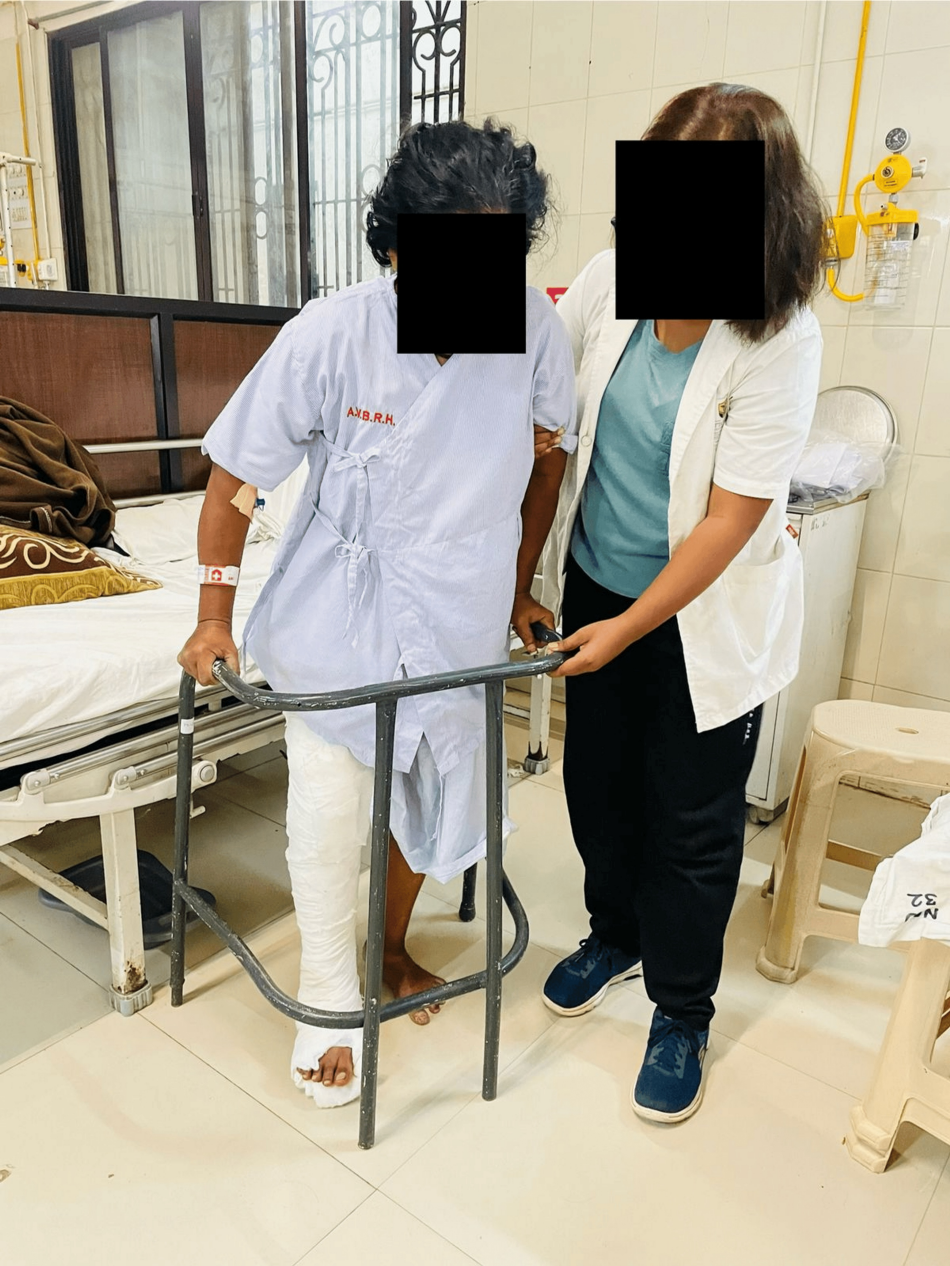
Weight-bearing ambulation

Figure 6 depicts partial weight-bearing ambulation being performed with the help of a walker.

Outcome measures

The pre- and post-intervention outcomes are shown in Table 10.

**Table 10:** Outcome measures VAS: 9.1 = severe; 4.3 = moderate IKDC-SKF, International Knee Documentation Committee subjective knee form; VAS, Visual Analogue Scale

Outcome measures	Pre-treatment	Post-treatment
VAS	9.1/10	4.3/10
Lower extremity function scale	11/80	36/80
IKDC-SKF	28.7/100	44.8/100

## Discussion

The management of MLKI combined with a medial condyle fracture is complex due to the required rehabilitation efforts, emphasizing the importance of early and comprehensive physiotherapy for recovery outcomes. MLKIs, including ACL, PCL, and collateral ligaments, can cause significant functional impairment and an increased risk of post-traumatic osteoarthritis. This patient’s postoperative physical therapy had a positive response; through targeted exercises and pain management techniques, they were able to significantly reduce pain, improve ROM, and build muscle strength. These positive outcomes demonstrate the effectiveness of a well-coordinated rehabilitation approach in addressing complex multi-ligament injuries, ultimately improving functional recovery and quality of life for patients. Recovering from MLKIs, especially when combined with a tibial medial condyle avulsion fracture, is a tough challenge that demands a well-rounded approach. These injuries usually involve several important ligaments, which makes the treatment process quite complex. Both the surgery and the rehabilitation plan must be carefully formed and managed to give patients the best chance of achieving a full recovery [16]. Aherrao et al., in their study, concluded that a postoperative physiotherapy plan to reduce pain, reduce swelling, and increase functional mobility is formed after ORIF surgery to improve the quality of life of the patient [17]. Lang et al., in a retrospective case series, have displayed a decrease in pain as well as increased scores for lower limb function, which suggests that a structured exercise program was effective [18]. Li et al., in a prospective study with 95 patients, concluded that patients with multiple knee injuries would benefit from a good program of rehabilitation. It has also been noted that early mobilization and progressive strength exercises reduce the extent of long-term disability and enhance recovery [19]. The outcomes of this study are thoroughly aligned with plenty of other studies, which state that a properly formed physical therapy plan is to cure acute knee injuries and improve patients’ recovery. Hunter et al. concluded the need for a program is to increase the intensity of exercise to reduce pain and swelling, which is helpful in recovery. Continuing to learn and develop this rehabilitation program should be a goal for the future to restore the quality of life for patients [20]. Khatri et al. concluded that after stable lag screw fixation of PCL tibial bone avulsion, it gives good clinical results. With minimal dissection, the posteromedial technique provides adequate exposure for screw fixation. Arthrofibrosis is avoided, and a good ROM is achieved with early rehabilitation compared to cast immobilization [21].

## Conclusions

The treatment of MLKI highlights the need for a careful and flexible approach to rehabilitation, especially when there are concurrent ACL and PCL avulsion fractures. According to the case study, early and focused physical treatment is essential for reducing pain, decreasing swelling, and regaining function. The rehabilitation approach greatly improves recovery results by combining pre-operative education, pain management, and postoperative strengthening and mobility exercises. This physiotherapy intervention not only treats acute functional deficits but also gets the patient ready for long-term stability and reduces the chance of falls. In the end, getting the best possible recovery and improving the general quality of life depend greatly on helpful physical treatment customized to the particular needs of multi-ligament injuries.

## References

[1] Ali AA, Abdelwahab MB (2019). Short-term outcome of multi-ligament knee injury among Sudanese patients. Open Access Maced J Med Sci.

[2] Nyland J, Mattocks A, Kibbe S, Kalloub A, Greene JW, Caborn DN (2016). Anterior cruciate ligament reconstruction, rehabilitation, and return to play: 2015 update. Open Access J Sports Med.

[3] Arthur JR, Haglin JM, Makovicka JL, Chhabra A (2020). Anatomy and biomechanics of the posterior cruciate ligament and their surgical implications. Sports Med Arthrosc Rev.

[4] Varelas AN, Erickson BJ, Cvetanovich GL, Bach BR Jr (2017). Medial collateral ligament reconstruction in patients with medial knee instability: a systematic review. Orthop J Sports Med.

[5] Olewnik Ł, Gonera B, Kurtys K, Podgórski M, Polguj M, Topol M (2019). A proposal for a new classification of the fibular (lateral) collateral ligament based on morphological variations. Ann Anat.

[6] Thiagarajan P, Ang KC, Das De S, Bose K (1997). Ipsilateral knee ligament injuries and open tibial diaphyseal fractures: incidence and nature of knee ligament injuries sustained. Injury.

[7] Skendzel JG, Sekiya JK, Wojtys EM (2012). Diagnosis and management of the multiligament-injured knee. J Orthop Sports Phys Ther.

[8] Vaidya R, Roth M, Nanavati D, Prince M, Sethi A (2015). Low-velocity knee dislocations in obese and morbidly obese patients. Orthop J Sports Med.

[9] Fanelli GC, Orcutt DR, Edson CJ (2005). The multiple-ligament injured knee: evaluation, treatment, and results. Arthroscopy.

[10] Sabesan VJ, Danielsky PJ, Childs A, Valikodath T (2015). Multiligament knee injuries with associated tibial plateau fractures: a report of two cases. World J Orthop.

[11] Pardiwala DN, Subbiah K, Thete R, Jadhav R, Rao N (2021). Multiple ligament knee injuries: clinical practice guidelines. JASSM.

[12] Levy BA, Dajani KA, Whelan DB (2009). Decision making in the multiligament-injured knee: an evidence-based systematic review. Arthroscopy.

[13] Iliadis DP, Bourlos DN, Mastrokalos DS, Chronopoulos E, Babis GC (2016). LARS artificial ligament versus ABC purely polyester ligament for anterior cruciate ligament reconstruction. Orthop J Sports Med.

[14] Nyland J, Brand E, Fisher B (2010). Update on rehabilitation following ACL reconstruction. Open Access J Sports Med.

[15] Ateschrang A, Ahmad SS, Stöckle U, Schroeter S, Schenk W, Ahrend MD (2018). Recovery of ACL function after dynamic intraligamentary stabilization is resultant to restoration of ACL integrity and scar tissue formation. Knee Surg Sports Traumatol Arthrosc.

[16] Jones M, Ball S, Williams A, Borque K (2020). Multi-ligament knee injuries in elite athletes: return to play rates, timing, and complications. Orthop J Sports Med.

[17] Aherrao S, Phansopkar P, Tikhile P (2024). The integral role of physiotherapy in optimizing movement and function in a case of polytrauma: a case report. Cureus.

[18] Lang PJ, Feroe A, Franco H, Hussain ZB, Tepolt FA, Kocher MS (2023). Outcomes of operative management of multi-ligament knee injuries in an adolescent population: a retrospective case series. J Pediatr Orthop.

[19] Li T, Xiong Y, Zhang Z (2021). Results of multiple ligament reconstruction after knee dislocation--a prospective study with 95 patients and minimum 2-year follow up. BMC Musculoskelet Disord.

[20] Hunter CW, Deer TR, Jones MR (2022). Consensus guidelines on interventional therapies for knee pain (STEP guidelines) from the American Society of Pain and Neuroscience. J Pain Res.

[21] Khatri K, Sharma V, Lakhotia D, Bhalla R, Farooque K (2015). Posterior cruciate ligament tibial avulsion treated with open reduction and internal fixation through the burks and schaffer approach. Malays Orthop J.

